# Endoscopic Injection of Mitomycin C for the Treatment of Pharyngoesophageal Stenosis Refractory to Endoscopic Treatment with Dilatation in Patients Treated for Head and Neck Cancer

**DOI:** 10.1155/2018/5428157

**Published:** 2018-11-12

**Authors:** Carla Cristina Gusmon-Oliveira, Yeda Mayumi Kuboki, Gustavo Andrade de Paulo, Marcelo Simas de Lima, Ricardo Sato Uemura, Bruno Costa Martins, Luciano Lenz Tolentino, Adriana Vaz Safatle-Ribeiro, Marco Aurelio Kulcsar, Ulysses Ribeiro, Fauze Maluf-Filho

**Affiliations:** Department of Gastroenterology, Endoscopy Unit of the Cancer Institute of São Paulo-ICESP, Brazil

## Abstract

**Background:**

Management of pharyngoesophageal stenosis (PES) in patients after head and neck cancer (HNC) treatment remains a challenge. It is not uncommon that PES is refractory to dilation sessions. This study aimed at evaluating the efficacy of Mitomycin C (MMC) endoscopic injection for the treatment of refractory pharyngoesophageal stenosis.

**Patients and methods:**

This is a prospective study in patients with dysphagia following head and neck cancer treatment, without evidence suggestive of tumor recurrence, and refractory to endoscopic treatment. These patients were submitted to endoscopic dilation of the stenotic segment with thermoplastic bougies, followed by injection of MMC. We repeated the endoscopic sessions every three weeks.

**Results:**

From January 2015 to May 2015, we treated 13 patients with PES. Three patients were initially enrolled in the study for refractory stricture. We observed adverse events in all of them, with intense neck pain and ulcer development, justifying the interruption of the trial.

**Conclusion:**

The repeated injection in the short interval of MMC in refractory PES is not recommended, because it resulted in serious adverse events.

## 1. Introduction

Management of pharyngoesophageal stenosis (PES) in patients after head and neck cancer (HNC) treatment remains a challenge [1].

The etiology of PES in this group of patients is probably multifactorial, being associated with surgical manipulation, ischemic effects of radiotherapy, and even cancer recurrence [2]. It is estimated that up to 50% of patients treated for advanced HNC will present some degree of dysphagia. PES is a frequent cause of posttreatment dysphagia of HNC patients. Although the exact prevalence is unknown, several retrospective series estimate that upper cervical stricture is present in 1% to 23% of the cases [3].

The initial approach to PES is endoscopic dilation with a reported success rate ranging from 76% to 96% [4]. In patients with refractory strictures to dilation sessions, endoscopic corticosteroid injection, such as triamcinolone, is usually added to the dilation sessions, which may increase the success of the endoscopic treatment [1, 5]. A relatively small group of patients will be refractory to the association of dilation and corticosteroid injection treatment. Metal stents may be considered in this scenario, but in PES, this approach is limited by cervical pain and foreign body sensation [6]. Surgical reconstruction for PES refractory to endoscopic treatment is the last therapeutic option [1]. In the previous radiotherapy, eventual reconstruction with cutaneous flaps makes a surgical manipulation of the cervical region a real challenge, with increased adverse event rate, including restenosis [1]. Because of such difficulties, this prospective study aimed at evaluating the efficacy of Mitomycin C (MMC) endoscopic injection for the treatment of pharyngoesophageal stenosis in patients who were treated for HNC refractory to endoscopic dilation treatment. There are descriptions of the use of Mitomycin C for scar prevention after ophthalmologic and otorhinolaryngological interventions [7]. Its use in endoscopy has been previously described for the treatment of laryngeal and tracheal stenosis [1, 8] as well as refractory esophageal stenosis [4].

Mitomycin C is a substance isolated from the bacterium Streptomyces caespitosus, which is used as a chemotherapeutic agent and has antiproliferative effects on fibroblasts, reducing fibroblast proliferation and collagen formation. Some studies demonstrated the ability to decrease fibroblast activity and consequent scar formation [4, 9] after the application of Mitomycin C on culture of fibroblasts in low concentrations (0.1 to 0.4 mg/ml) for 5 to 10 minutes, affecting their proliferation for over 3 weeks.

## 2. Patients and Methods

This prospective study started on January 2015 and was meant to include 10 consecutive patients with PES refractory to endoscopic dilation. The protocol was approved by the research ethics committee of the Cancer Institute of the University of São Paulo (ICESP). We included patients ≥18 years old, with dysphagia due to a stricture following head and neck cancer treatment, without endoscopic or radiological evidence suggestive of tumor recurrence, and refractory to endoscopic treatment, who accepted participating in the study and signed the consent form. The exclusion criteria were endoscopic or radiological evidence suggestive of tumor recurrence.

Patients were considered refractory to endoscopic treatment when we observed the recurrence of the stricture and dysphagia after two consecutive endoscopic dilation treatments to 15 mm.

### 2.1. Methods

We adopted the validated dysphagia scoring system [10]. We assessed the degree of dysphagia at the patient's inclusion and at the end of the treatment (Table 1). Standard gastroscopes (GIF-Q180 and GIF-H180; Olympus Optical Co., Ltd., Tokyo, Japan) and Savary-Gilliard dilators (Wilson-Cook Medical Inc., Winston-Salem, NC) were used.

**Figure 1:**
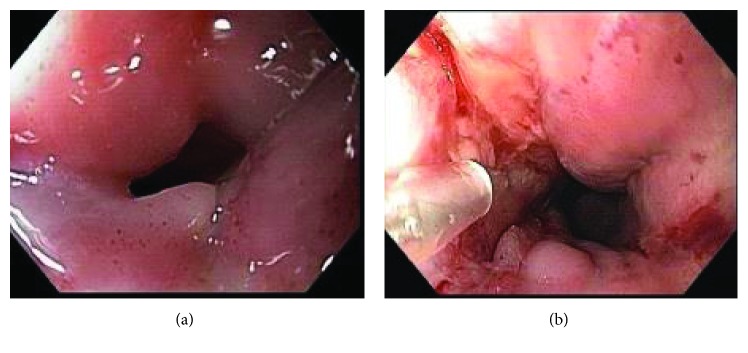
Pharyngoesophageal stricture. (a) Pharyngoesophageal stricture before dilation. (b) Injection of MMC after dilation.

All patients underwent the procedures (endoscopy and MMC injections) under intravenous sedation performed by nonanesthesiologists. A combined sedation with fentanyl, midazolam, and propofol was used. All patients were monitored with electronic assessment of blood pressure, heart rate, and pulse oximetry and visual assessment of ventilator activity, level of consciousness, and discomfort. The technique of MMC administration included dilation of the stenotic segment with thermoplastic bougies, followed by the injection of 3 mg of MMC, divided into 4 aliquots of 0.75 mg and injected in the four quadrants at the stenosis level [11] (Figure 1).

The same physician performed all therapy, and the medication was applied in the normal mucosa adjacent to the lacerated mucosa. For the included patients, the study protocol was to perform four consecutive sessions with intervals of 3 weeks of dilation associated with MMC injection in each session. The aim of the four sessions of endoscopic treatment was to reach a diameter of 15 mm, always respecting the rule of 3 for dilation [12]. All patients received proton pump inhibitor at a dose of 20 mg orally every 12 hours up to 4 weeks after the last application of MMC. They were oriented to take a soft diet and to use analgesics if necessary.

## 3. Results

From January 2015 to May 2015, we treated 13 patients with PES; from them, three patients (2 male) were initially enrolled in the study, with mean age of 63 years (61–68 years). The MMC was dispensed by ICESP pharmacy; so, for logistic reasons, we performed the endoscopic sessions of MMC injection for the 3 included patients in the same day.

All patients had T4 stage squamous cell carcinoma of the upper aerodigestive tract. Two of them were submitted to total laryngectomy associated with postoperative radiotherapy. The third patient was treated exclusively with chemoradiation. All three patients developed strictures of the pharyngoesophageal segment refractory to repeated sessions of dilation. The number of dilation sessions before inclusion was 10, 6, and 8, respectively (Table 2). Four years (50 months, range from 24 to 96 months) was the mean time elapsed between the initial cancer treatment and the dilation associated with MMC.

**Figure 2:**
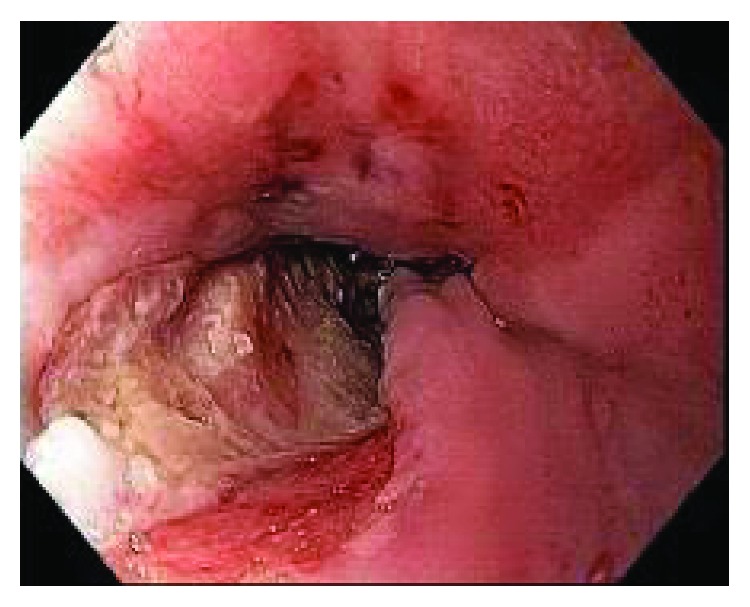
Ulcer development in the pharyngoesophageal transition after MMC injection.

The first patient underwent three sessions of dilation and MMC injection. She developed a symptomatic, 3 cm large, painful ulcer in the pharyngoesophageal transition after the third injection, and then the MMC injection was interrupted (Figure 2). The ulcer was completely healed two months later. The biopsy was negative to neoplasia. Her grade of dysphagia improved from 2 to 1 at the end of the treatment; however, she required more sessions of dilations because of a persistent stricture.

**Figure 3:**
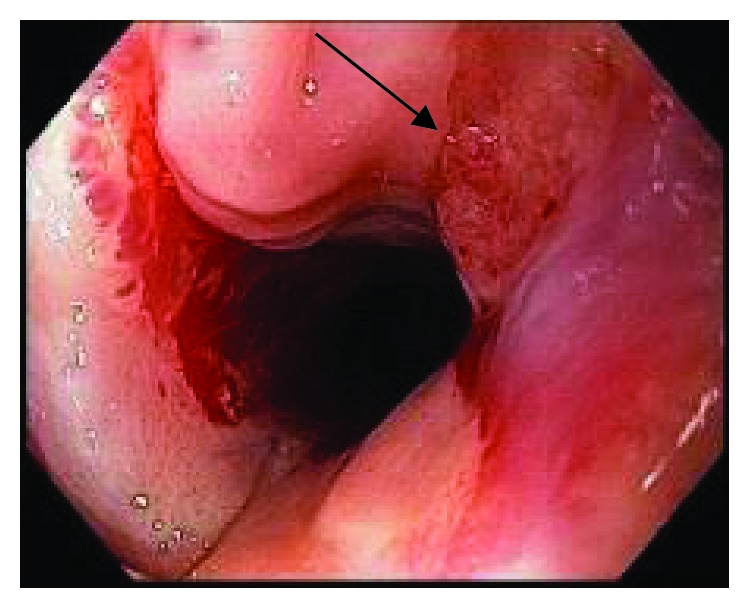
Irregular mucosal area in hypopharynx after just one session of MMC: early neoplasia.

The other two patients who underwent two sessions of MMC injection also presented serious complications. One developed a small ulcer and an irregular mucosal area in hypopharynx after just one session. He was submitted to a second MMC injection. However, the histology of the ulcer proved positive for malignancy (Figure 3). During the follow-up, the patient became more symptomatic, with intense neck and swallow pain. The ulcer was completely healed 9 months after the first injection. This neoplastic lesion was resected by ESD, and no evidence of neoplasia was present in the 30-month follow-up. He remained with a grade 3 dysphagia caused by the PES.

**Figure 4:**
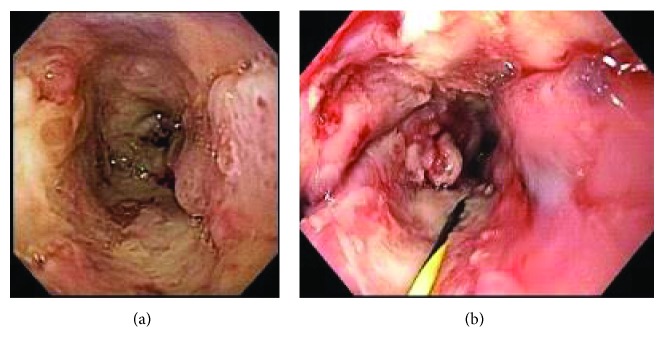
Bleeding ulcer post-MMC injection. (a) Large ulcer post-MMC injection. (b) Bleeding ulcer.

The third patient had a complex PES with dysphagia for liquids and gastrostomy tube for nutritional support. He underwent two sessions of dilation associated with MMC injection. He developed intense pain and grade 4 dysphagia. At this point, he tolerated exclusively gastrostomy feeding, possibly explaining the maintenance of weight. At endoscopy, an ulcer of 4 cm was identified. This ulcer was the source of four episodes of bleeding confirmed by endoscopy evaluation (Figure 4). The ulcer was biopsied and was negative for malignancy. The last bleeding episode occurred 5 months after the MMC injection, causing patient's death.

All the patients developed local ulcers due to MMC injection, causing intense pain and also intensification of the dysphagia. None of them completed the 4 (four) proposed sessions.

No sedation related adverse events were observed.

The study was interrupted due to the adverse events described above, meaning that the remaining eligible 10 patients were not included. The mean follow-up was 24 months (5–34). Two patients who are still in follow-up have strictures that do not allow the passage of the standard endoscope, still requiring repeated dilation sessions. They both tolerate soft diet without weight loss.

## 4. Discussion

In this prospective study with MMC injection, we observed adverse events in three included patients, with intense neck pain and ulcer development, justifying the interruption of the trial. Moreover, one patient died from a bleeding ulcer at the injection site 5 months after the first injection.

During the 30-month follow-up period, only one patient had a sustained improvement of the grade of dysphagia, from 3 to 1, despite stricture persistence.

Our results demonstrate that injection of MMC is associated with a high rate of adverse events, as demonstrated by Wu et al. in the animal studies in the past [13].

In a recently published systematic review, 24 studies of refractory esophageal strictures treated with MMC were included: 1 randomized controlled trial (40 patients), 3 prospective studies (44 patients), and 20 case reports or small case series. The majority of the publications (17 studies) enrolled only pediatric patients, corresponding to 70% of them. The most common etiology was caustic injury, with just 18% of the patients with anastomotic stricture and only 2 adult patients with radiation stricture [14]. Overall, 94% of 117 patients (pediatric and adults) included had a good response. From 37 adult patients, improvement of dysphagia was observed in all of them. Only one adverse effect was observed (0.7%) related as a “cutaneous sclerosis” and rash due to microperforation after MMC injection and extravasation of the substance to the superior mediastinum and to the skin of the upper chest and neck [15].

The literature review of MMC in the treatment of esophageal strictures in adults includes eight uncontrolled series adding to 70 patients. All reported significant improvement in dysphagia, a decrease in the number of sessions of endoscopic dilations, despite of the persistence of strictures in 25% of them [16].

We hypothesized that the discrepancy between our results and the results reported in the literature could be due to the MMC application to the affected area. The majority of studies reported topical application of MMC with a variety of application techniques, including cotton pledget, microporous balloon, and spraying technique. However, we found 4 studies with 32 adult patients submitted to MMC injection directly in the stricture after dilation [11, 17–19].

Zhang et al. and Gillespie et al. published the largest studies about the topical use of MMC in adults for esophageal stenoses [3, 19]. The patients enrolled had strictures due to anastomosis, radiation, and after ESD. The improvement of dysphagia, including decreased need of dilation and increased esophageal lumen, can possibly be explained by the fact that the patients were in the beginning of the treatment (less than five dilation sessions) or had not been treated yet. Bartel et al. included adult patients with five or more previous dilations and observed less favorable outcomes [16].

Our patients had complex strictures with multiple dilation sessions, which might have induced strictures less amenable to endoscopic treatment with MMC. On the other hand, the ischemic component induced by previous radiation might be involved in the serious adverse events observed in our series. In addition, the technique with repeated injection adopted in the present study might have caused a MMC accumulated effect. Another possible explanation for the severe adverse events is that during the MMC injection, we applied the drug in the normal mucosa adjacent to the lacerated mucosa, but we did not irrigate the mucosa with water or saline solution after the injection to wash any leaked drug. We are not sure about this leak, but it is not possible to exclude it. Previous results support MMC injection, but not with short intervals between sessions [11, 17–19].

Our study has several limitations, especially the lack of a control group and the small number of patients due to complications.

In conclusion, the repeated injection of MMC, 3 mg (4 aliquots of 0.75 mg), in refractory PES of patients treated from HNC resulted in serious adverse events with partial success in only one out of 3 patients. We argue against repeated MMC injection in short interval. Avoiding injection, giving preference for topical application could be a safe technique associated with a better outcome.

Another studies are necessary to answer questions and to establish the optimal strategy of the endoscopic use of MMC for the treatment of refractory PES.

## Figures and Tables

**Table 1:** Dysphagia scoring system [10].

0	Able to eat normal; no dysphagia
1	Able to shallow some solid foods
2	Able to shallow only semisolid foods
3	Able to shallow only liquids
4	Unable to eat; total dysphagia

**Table 2:** Baseline characteristics of patients with benign and refractory pharyngoesophageal strictures who underwent MMC injection.

	Age		Tumor	Cancer treatment	Time since treatment	Sessions of MMC	Grade of dysphagia before × after	Weight
Patient 1 V. F.	62	F	SCC larynx T4N2M0	Total laryngectomy + CRDT	8 years	3	2 × 1	The same
Patient 2 A. F.	68	M	SCC larynx T4N1M0	Total laryngectomy + CRDT	2 years	2	3 × 3	The same
Patient 3 I. G.	61	M	SCC hypopharynx and cervical esophagus T4bNxMx	CRDT	2, 5 years	2	3 × 4 GTT	The same (gastrostomy)

## Data Availability

The data used to support the findings of this study are included within the article.
